# An evaluation of access to health care services along the rural-urban continuum in Canada

**DOI:** 10.1186/1472-6963-11-20

**Published:** 2011-01-31

**Authors:** Lyn M Sibley, Jonathan P Weiner

**Affiliations:** 1 Institute for Clinical Evaluative Sciences, Sunnybrook Health Sciences Centre, 2075 Bayview Avenue, Toronto ON, M4N 3M5 Canada; 2 Health System Performance Research Network, Department of Health Policy, Management, and Evaluation, University of Toronto, 155 College Street - 425, Toronto ON, M5T 3M6 Canada; 3 Department of Health Policy and Management, Bloomberg School of Public Health, Johns Hopkins University, 624 N. Broadway, Baltimore MD, 21205 USA

## Abstract

**Background:**

Studies comparing the access to health care of rural and urban populations have been contradictory and inconclusive. These studies are complicated by the influence of other factor which have been shown to be related to access and utilization. This study assesses the equity of access to health care services across the rural-urban continuum in Canada before and after taking other determinants of access into account.

**Methods:**

This is a cross-sectional study of the population of the 10 provinces of Canada using data from the Canadian Community Health Survey (CCHS 2.1). Five different measures of access and utilization are compared across the continuum of rural-urban. Known determinants of utilization are taken into account according to Andersen's Health Behaviour Model (HBM); location of residence at the levels of province, health region, and community is also controlled for.

**Results:**

This study found that residents of small cities not adjacent to major centres, had the highest reported utilisation rates of influenza vaccines and family physician services, were most likely to have a regular medical doctor, and were most likely to report unmet need. Among the rural categories there was a gradient with the most rural being least likely to have had a flu shot, use specialist physicians services, or have a regular medical doctor. Residents of the most urban centres were more likely to report using specialist physician services. Many of these differences are diminished or eliminated once other factors are accounted for. After adjusting for other factors those living in the most urban areas were more likely to have seen a specialist physician. Those in rural communities had a lower odds of receiving a flu shot and having a regular medical doctor. People residing in the most urban and most rural communities were less likely to have a regular medical doctor. Those in any of the rural categories were less likely to report unmet need.

**Conclusion:**

Inequities in access to care along the rural-urban continuum exist and can be masked when evaluation is done at a very large scale with gross indicators of rural-urban. Understanding the relationship between rural-urban and other determinants will help policy makers to target interventions appropriately: to specific demographic, provincial, community, or rural categories.

## Background

Canadian policy makers have long struggled with how best to provide timely and appropriate access to high quality health care to all Canadians especially those who live in the rural areas of the country. Universal health insurance coverage has eliminated many barriers to receiving appropriate, high quality health care, however geography remains a potential barrier to access.

Considering the attention that is placed on access to health care services it is surprising the degree of ambiguity in defining it. In the United States access is often synonymous with health insurance. This implies that once everyone has insurance there will be some degree of equality in the utilization of health care services. How, then is access defined in an environment, such as Canada, where everyone has health insurance? The Canada Health Act (CHA) lists access as one of its five main tenants stating that "persons must have reasonable and uniform access to insured health services, free of financial or other barriers. No one may be discriminated against on the basis of such factors as income, age, and health status [1]." While there is general agreement about the value of this principle, there is a great deal of national discourse about how this should be interpreted, applied, and evaluated [2-4].

Andersen has operationalized a definition of access to be used in health services research. He states that access is "the actual use of personal health services and everything that facilitates or impedes the use of personal health services [5]." In this definition, access consists of two components "the use of health services" and "everything that facilitates or impedes the use." It is this definition on which the Health Behaviour Model, the conceptual framework for this study is based.

An important concept in the evaluation of access is equity. Health care services are equitably distributed when health status and demographic indicators of health status are the strongest predictors of who uses health care [6]. When evaluating the degree of equity, indicators of need are considered; in an equitable system those with equal need will have equal utilization rates (horizontal equity) and those with less need will have lower utilization rates (vertical equity) [7].

Intuitively it is expected that rural populations have reduced access to health care services compared to urban populations. However studies comparing access of rural and urban populations have been contradictory and inconclusive. Whether or not differences between rural and urban populations are observed depends on the measure of access that is assessed, how rural-urban status is classified, and what other factors, such as geographic location beyond rural-urban, are taken in to account [8].

Many of the studies that compare access variables between rural and urban communities, use a dichotomous variable for rural-urban. This does not account for varying degrees of rurality of communities. Studies that use a binary indicator of rural-urban are less likely to observe a difference in access measures than studies that evaluate communities along a more specific continuum of rural-urban. A more specific scale acknowledges that large towns that are near major cities are very different from small towns in remote locations.

Studies that control for geographic location, or that focused on a specific region had differing results, than large scale studies that do not account for region, state, province, etc. There is a burgeoning body of literature on the effect of place of residence on health [9,10] and although not growing as quickly, there is also a body of inquiry into the effects of place of residence on health services utilisation [11-14]. Where people live has been shown to be associated with their health status, health behaviours, and their utilization of health care services.

In order to get a complete understanding of access to health care along the rural-urban continuum this study uses five different measures of access that reflect both potential and realized access; uses a five level indicator of rural-urban status; and accounts for location of residence at the levels of province, health region, and community. The first objective of this study is to compare access to health care services in Canada across the rural-urban continuum. The second objective is to determine how much of the variation in access across the rural-urban continuum remains once other determinants are accounted for.

## Methods

This is a cross-sectional study of the population of the 10 provinces of Canada using data from the Canadian Community Health Survey (CCHS 2.1) conducted in 2003. Five different measures of access and utilization are compared across the continuum of rural-urban. Known determinants of utilization are taken into account according to Andersen's Health Behaviour Model (HBM) [15], and location of residence at the levels of province, health region, and community.

### Data Source

The CCHS is a very comprehensive national population survey aimed at describing the health and health services experiences of Canadians. The survey was conducted by Statistics Canada in collaboration with the federal Ministry of Health, the territorial and provincial Ministries of Health, and the Canadian Institute for Health Information. The CCHS survey questionnaire was administered using computer-assisted interviewing in-person, when possible, and by telephone when a face-to-face interview was not feasible [16].

Based on the available resources and the desire to produce reliable statistics at the health region level, CCHS 2.1 aimed to include 133,700 respondents. The sample was divided among the provinces based on the population and the number of health regions. Each province's sample was then distributed among the health regions proportionally to the square root of the population in each HR. The survey response rate was 80.7%. Individuals that were sampled but did not respond to the survey were accounted for in the survey weights [16].

Statistics Canada estimates that the survey is representative of approximately 98% of the Canadian population aged 12 and older. The 2% that are not represented include those residing on Indian Reserves or Crown lands, in institutions, in certain remote areas of the territories, or who are full-time members of the Canadian Armed Forces [16].

This study is based on data collected from adults aged 20 and older and lived in one of the 10 provinces of Canada. The two percent of survey subjects who had their responses given by proxy -- either because they were not available or they were unable to answer because of language or poor health -- were excluded from this study.

### Study Variables

The five survey derived outcome variables used in this analysis are:

• having had an influenza vaccination in the previous two years;

• having seen a family physician in the previous 12 months;

• having seen a specialist physician (other than an eye doctor) in the previous 12 months;

• self-perceived unmet need in the previous 12 months; and

• having a regular medical doctor.

These outcomes were chosen because they reflect health care access from a broad range of sectors, including primary and secondary care; preventative and therapeutic interventions; and utilization and factors that facilitate utilization. These variables were also expected to show adequate variation across the population.

Having had an influenza vaccination in the last two years is an outcome measure of having received high quality primary health care. The 2002-2003 influenza season National Advisory Committee on Immunization recommendations to Health Canada state that people at risk of influenza-related complications be immunized: people with chronic conditions, residents of nursing homes, people age 65+. They also recommend that people who are in close contact with these at risk groups, such as care givers and health professionals be immunized. Finally, they recommend that healthy people who wish to avoid getting the flu be immunized [17].

Self-perceived unmet need occurs when an individual feels that they required health care services but for one reason or another they did not receive them. Specifically respondents are asked "During the past 12 months, was there ever a time when you felt that you needed health care but didn't receive it?" The benefit of using this outcome is that it does not rely on respondents seeing a physician. It provides some insight into the health care needs of those who have not seen a physician.

There are two different variables used to assess the use of physician services: having had one or more consultations with a family physician, and having had one or more consultations with a specialist physician. These were based on responses to the following questions.

1. Not counting when you were an overnight patient, in the past 12 months, how many times have you seen, or talked on the telephone, about your physical, emotional or mental health with: ... a family doctor, or general practitioner?

2. Not counting when you were an overnight patient, in the past 12 months, how many times have you seen, or talked on the telephone, about your physical, emotional or mental health with: ... any other medical doctor (such as a surgeon, allergist, orthopaedist, gynaecologist or psychiatrist)?

It is estimated that the Canadian National average number of physician visits among those who had a visit in 2003 was 5.2 for family physician visits, and 3.2 for specialist visits [18].

The final outcome measure was having a regular medical doctor. This measure of health behaviour is also an enabling resource in the health behaviour model. The variable was derived from the response to the questions "Do you have a regular medical doctor?" Often this variable is referred to as usual source of care, however in this case the survey explicitly referred to a medical doctor.

In his previous works Andersen referred to the utilization of health services as realized access and to factors that facilitate the use of services as potential access [15]. To avoid confusion in this paper if the word access is used it will mean both potential and realized access in general, otherwise the specific component will be referred to directly.

The indicator used to represent communities on the rural-urban continuum is the Statistical Area Classification (SAC) [19] as recommended by Statistics Canada in the Rural and Small Town Canada Analysis Bulletin [20]. The basis of this definition is Census Subdivisions which are legislatively determined municipalities or equivalents. According to the definition each Census Subdivision with a population less than 10,000 is categorized as rural or small town. These rural and small towns are further classified into Metropolitan Influence Zones (MIZs) which are determined by the percentage of the community population that commutes to a city or urban centre for employment. Urban municipalities are classified as Census Agglomerations (CAs), small urban centres with between 10,000 and 100,000 people, or Census Metropolitan Areas (CMAs), urban centres with 100,000 people or more [19,20]. The Statistical Area Classification is given in Table 1 along with the population of each category [21,22].

**Table 1:** Statistical Area Classification Hierarchy (2001)[20,21]

	Category	Description	% of Canadian Population
**Urban**			

	Census Metropolitan Areas (CMA)	One or more adjacent Census Subdivisions (CSDs) situated around a major urban core (population ≥ 100,000).	64.3

	Census Agglomerations (CA)	One or more adjacent CSDs situated around a major urban core (population ≥ 10,000).	15.1

**Rural**			

	Strong Metropolitan Influenced Zone (MIZ)	A CSD where more than 30% of residents commute to work in an urban core (population < 10,000).	5.1

	Moderate MIZ	A CSD where between 5% and 30% of residents commute to work in an urban core (population < 10,000).	7.6

	Weak MIZ	A CSD where between 0% and 5% of residents commute to work in an urban core (population < 10,000).	6.6

	No MIZ	A CSD where forty or fewer residents commute to work in an urban core (population < 10,000).	1.1

Geographic location is assessed at the level of province, health region, and Consolidated Census Subdivision. Health care services are administered provincially in Canada and each province is divided into a number of health regions for administrative purposes. There are 126 health regions in the country and the number of health regions per province varies from one in each of the territories to 37 in the province of Ontario. Consolidated Census Subdivisions (CCSs) are municipalities or adjacent economically dependent communities [19].

The other independent predictor variables were selected based on Andersen's Health Behaviour Model (HBM) and other research on determinants in Canada [15]. These predictors were identified as components of: need, predisposing characteristics, and enabling factors.

Two measures of need are used in this study: the presence of chronic conditions and self-rated health status. The measure of chronic conditions indicates whether subjects have zero, one, or two or more chronic conditions. In the questionnaire respondents were given a list of chronic conditions (Table 2) that was preceded by the instruction "Now I'd like to ask about certain chronic health conditions which you may have. We are interested in 'long-term conditions' which are expected to last or have already lasted 6 months or more and that have been diagnosed by a health professional." The number of conditions reported by each person was summed. Self-rated health status is a widely used measure of need and has been shown to be strongly related to utilization of health care services. This measure has the five categories excellent, very good, good, fair, and poor which are rated by survey respondents in response to the question "In general, would you say your health is?"

**Table 2:** Chronic Conditions

Asthma	Cataracts
Fibromyalgia	Glaucoma
Arthritis or rheumatism	Thyroid condition
High blood pressure	Chronic fatigue syndrome
Migraine headaches	Multiple chemical sensitivities
Diabetes	Schizophrenia
Epilepsy	Mood disorder
Heart Disease	Anxiety disorder
Cancer	Other developmental disorder
Stomach or intestinal ulcers	Eating disorder
Effects of stroke	Chronic bronchitis
Bowel disorder/Crohn's or colitis	Emphysema of COPD
Alzheimer's disease or other dementia	Other long-term health conditions

Predisposing characteristics describe an individual's propensity to use health care services. They are generally demographic factors that are related to utilisation and are not easily altered. The predisposing variable used in this study were: sex, age, marital status, educational attainment, and ethnic origin.

Enabling resources are the means that individuals have available to them for the use of health care services. These factors are generally more mutable than predisposing characteristics, as they include such things as insurance and availability of physicians. Other enabling factors that are not as easily modified, at least in the short term, are household income, and employment status. The enabling factors used in this study were: having a regular medical doctor, income adequacy, having pharmaceuticals insurance, and occupation class.

Note that the variable "has a regular medical doctor" is included as both an enabling resource (independent variable) and a measure of access (dependent variable). Having a regular medical doctor is a resource that enables the use of care, however having a regular medical doctor to provide care when needed is an indicator of access.

Greater detail on each of the variables can be found in the survey documentation [16].

### Missing Data

For each question or category of questions there is a percentage of respondents for whom the question was not appropriate, or who were unable or unwilling to answer. Comparisons of the rates with and without the missing values revealed very little difference in most cases. The only notable difference was in income adequacy, where a response is not given for 12.3% of the theoretical population. Dropping these records from the analysis would mean dropping a substantial percentage of the sample and may lead to misleading conclusions. Because income adequacy is an important variable to consider as a determinant of health services utilization a category for "Not Stated" was included in the analysis. Because a separate coefficient/odds ratio was be assigned to this category it is assumed that these people have something in common with each other, and that the non-responders are not randomly distributed through the population.

### Analytical Approach

The analytical approach to address the first objective was to calculate unadjusted odds ratios to compare each access measure across the rural-urban continuum. To address the second objective multilevel logistic regression models were developed that included the HBM variables and accounted for location at the levels of province, health region, and without CCS. The odds ratios for the six levels of rural-urban were evaluated at each iteration of the model. To build the logistic regression models and decide on the variables used the steps outlined by Hosmer and Lemeshow were followed [23].

Multilevel models were developed following the methods described by Luke and Snijders [24,25]. The observed variance that is attributed to health region and CCS was reported as intraclass correlation coefficients (ICCs)[24], and median odds ratio (MOR). The ICC is interpreted as the proportion of the overall variance that is attributable to a given level of the data hierarchy [24]. The median odds ratio (MOR) is used to quantify the heterogeneity between place of residence on the odds ratio scale so that it can be compared with the odds ratios given for the fixed effects variables [26-28].

As with any complex survey design the subjects in this study were not a simple random sample of the population. The CCHS used a multi-staged cluster design where each subject is assigned a weight indicating the number of individuals that they are meant to represent. The weights are used to derive meaningful estimates of population rates. A bootstrap re-sampling technique is used to estimate any sampling error in the survey when calculating the variances and confidence intervals [16].

## Results

The survey sample for this study includes 111,258 individuals aged 20 or older that lived in one of the ten Canadian provinces in 2003. After applying the sampling weights, the sample represents approximately 22.6 million people or 69.5% of the population of Canada.

The first objective of this study is to compare access to health care services in Canada across the rural-urban continuum. Table 3 and Table 4 compare the access measures across SAC. These results show that residents of Census Agglomerations, that is small cities not adjacent to major centres, had the highest reported utilisation rates of influenza vaccines and family physician services, were most likely to have a regular medical doctor, and were most likely to report unmet need. Among the rural categories there was a gradient with the most rural (no or weak Metropolitan Influence Zone [MIZ]) being least likely to have had a flu shot, use specialist physicians services, or have a regular medical doctor. Residents of the most urban centres (Central Metropolitan Area [CMA]) are more likely to report using specialist physician services.

**Table 3:** Distribution of Variables Across Rural-Urban Categories (% responding affirmatively)

Statistical Area Classification	Flu Shot	Unmet Need	Family Physician Visit	Specialist Physician Visit	Regular Medical Doctor
Urban - CMA^a^	35.9	11.7	78.5	29.4	85.2
Urban - CA	37.0	12.7	78.7	26.6	89.1
Rural - Strong MIZ	35.5	10.8	75.9	27.1	87.5
Rural - Moderate MIZ	32.7	10.2	76.2	24.6	83.4
Rural - Weak or No MIZ	30.5	11.2	77.4	22.8	83.9

^a ^CMA = Census Metropolitan Areas; CA = Census Agglomerations; MIZ = Metropolitan Influenced Zone;

**Table 4:** Unadjusted Univariate Logistic Regression -- Comparison of Access Measures by Rural-Urban. Odds Ratio (95% CI) Higher numbers indicate a higher likelihood of outcome

Statistical Area Classification	Influenza Vaccine	Self-Reported Unmet Need	Family Physician Consultation	Specialist Physician Consultation	Regular Medical Doctor
Urban - CMA^a^	0.95 (0.91, 1.00)^c^	0.91 (0.84, 0.97)^c^	0.99 (0.93, 1.05)	1.15 (1.09, 1.21)^d^	0.70 (0.65, 0.76)^d^
Urban - CA^b^	1.00	1.00	1.00	1.00	1.00
Rural - Strong MIZ	0.92 (0.84, 1.01)	0.83 (0.73, 0.95)^c^	0.85 (0.77, 0.95)^c^	1.03 (0.93, 1.13)	0.86 (0.74, 0.99)
Rural - Moderate MIZ	0.83 (0.77, 0.89)^d^	0.78 (0.70, 0.87)^d^	0.86 (0.79, 0.95)^c^	0.90 (0.83, 0.98)^c^	0.85 (0.75, 0.95)^c^
Rural - Weak or No MIZ	0.75 (0.70, 0.80)^d^	0.86 (0.78, 0.95)^c^	0.93 (0.86, 1.00)^c^	0.81 (0.76, 0.87)^d^	0.64 (0.58, 0.70)^d^

^a ^CMA = Census Metropolitan Area; CA = Census Amalgamation; MIZ = Metropolitan Influence Zone

^b ^Reference Category

^c ^Significant at p < 0.05

^d ^Significant at p < 0.001

The second objective of this study is to evaluate how much variation in access there is once other determinants are taken in to account. Multiple logistic regression models were built to assess factors that are related to each of the five access measures and to evaluate how much the variation across the rural/urban continuum changes once these factors are controlled for. These models are given in Additional file 1, Appendix B.

The two indicators of need, self-rated health status and chronic conditions, are very strong predictors of access and show a clear pattern across all of the access measures. The demographic variables of age and sex were strongly related to the access measures, with older people and women reporting higher levels of utilization. Women have a higher likelihood of using health care services, having a regular medical doctor and reporting unmet health care needs. Those aged 70 or higher are less likely to use specialist physician services than those in the younger age groups. People in the oldest age groups are also less likely to report having had unmet health care needs. Being married or equivalent increases the likelihood of a family or specialist physician visit and of having a regular medical doctor. Marital status is not related to having a flu shot or reporting unmet need.

Those with the highest level of educational attainment, post-secondary school graduation, were more likely to report having had a flu shot to have had a consultation with a physician; and to report having unmet health care needs. Those with the lowest level of education, less than secondary school graduation, had a significantly lower likelihood of having seen a specialist physician.

Respondents who had an ethnic origin other than white were more likely to report receiving an influenza vaccination and having a regular medical doctor; and were less likely to have consulted a family or specialist physician, and report unmet health care needs.

There is a relationship between income and access in this study. Those in the lowest quartile for household income were less likely to have had a flu shot, have seen a specialist or to have a regular medical doctor. They were more likely to report having unmet need and equally as likely to have seen a family physician. Having pharmaceutical insurance is positively related to all five access measures even though none of them are directly related to the purchase of pharmaceuticals. An unusual pattern is seen among people who were employed for only part of the year. These people were less likely to have a flu shot, more likely to report unmet need, more likely to see a family or specialist physician and less likely to have a regular medical doctor.

There is a great deal of variation by province in having received an influenza vaccine in the previous two years. The results of the survey show that residents of Ontario have the greatest odds of receiving a flu shot while residents of Newfoundland have the lowest odds. Variation by province is also revealed in physician utilisation with residents of Quebec and Ontario being less likely to report having consulted a family physician in the previous 12 months and residents of Quebec having a 36% higher likelihood of consulting a specialist physician. Residents of Nova Scotia have a greater than two-fold increase in their likelihood of having a regular medical doctor while residents of Quebec have a reduced odds of reporting having a regular medical doctor. The municipality (CCS) of residence is attributed with a moderate level of variation in each of the outcomes, most marked being having a regular medical doctor. There is only a very small amount of variation attributed to health region.

**Figure 1:**
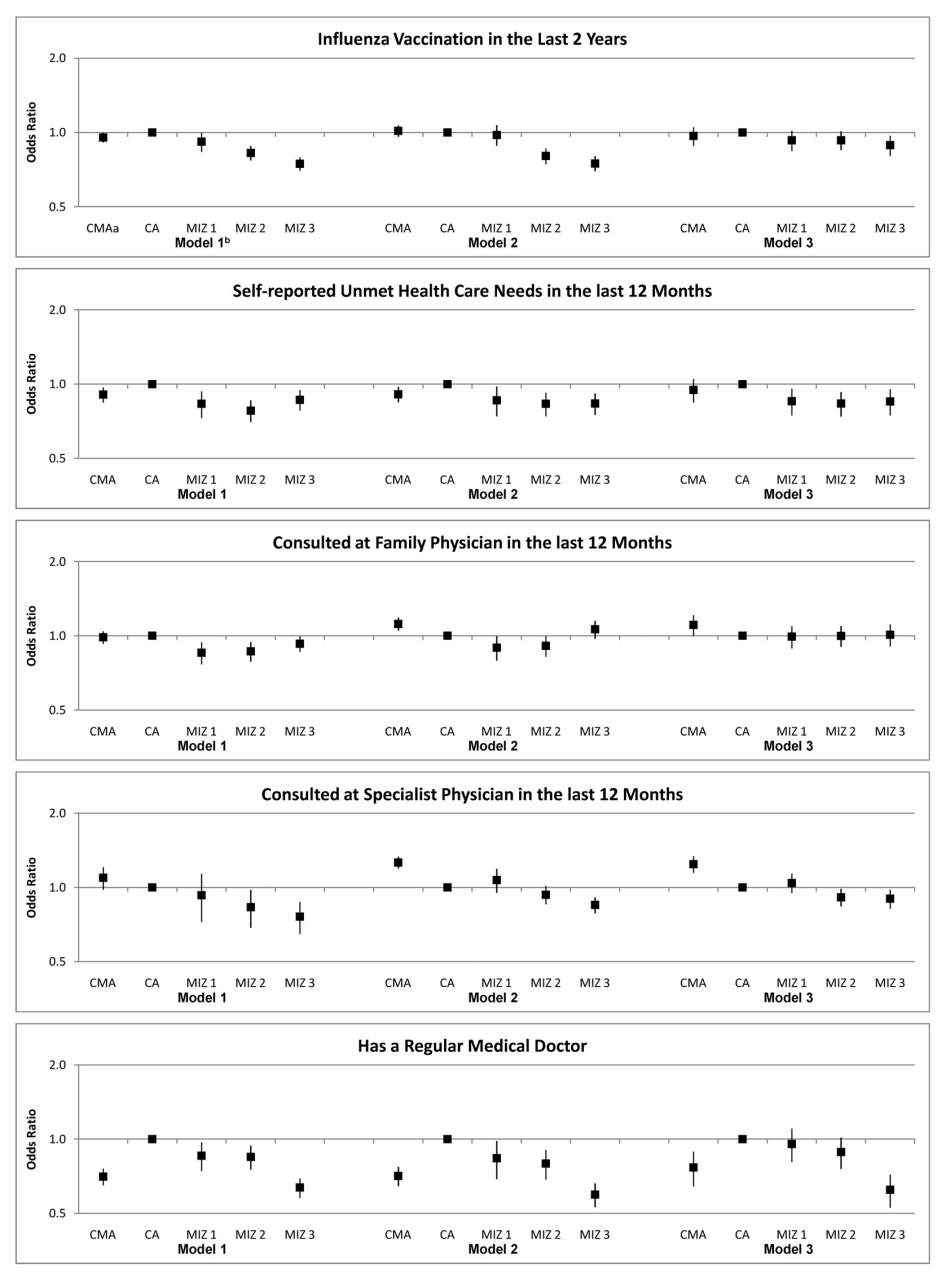
**Multilevel Logistic Regression Results for Rural-Urban Status and Place of Residence**. ^a ^CMA = Census Metropolitan Area; CA = Census Amalgamation; MIZ = Metropolitan Influence Zone ^b ^Model 1 = unadjusted model; Model 2 = model controls for chronic conditions, self-rated health status, age, marital status, educational attainment, ethnic origin, having regular medical doctor, income adequacy, having pharmaceuticals insurance, and occupation class; Model 3 = adjusted for variables in Model 2 as well as place of residence: province, health region, and Consolidated Census Subdivision.

Table 5 presents the adjusted odds ratios and confidence intervals for rural-urban status and place of residence. In order to assess the influence of the independent variables on access by rural-urban status Figure 1 compares the odds ratios for each rural-urban category unadjusted, adjusting for variables in the HBM, and adjusting for both HBM variables and location of residence. There is very little change in the odds of having received a flu shot after adjusting for the HBM variables. After adjustment for place of residence the differences are diminished with only those living in the most rural communities having a lower odds of receiving a flu shot. After adjusting for place there is no longer a statistically significant difference in the odds of reporting unmet health care needs between the two urban categories. Those in rural areas are less likely to report unmet need even after adjusting for HBM and place of residence. Most of the variation in family physician consultations is accounted for by need, predisposing characteristics, and enabling factors. Taking the effect of place into account eliminates any difference for those in the most urban cities. Those living in urban centres were more likely to have seen a specialist physician; this difference is not accounted for by HBM variables or place of residence. The lower rate of specialist utilisation by those living in the most rural areas is accounted for by province, health region, and community of residence. Those living in the most urban and the most rural are least likely to have a regular medical doctor regardless of the HBM factors or place of residence.

**Table 5:** Adjusted^a ^Multilevel Logistic Regression -- Comparison of Access Measures by Rural-Urban Status Odds Ratio (95% CI) Higher numbers indicate a higher likelihood of outcome

Statistical Area Classification	Influenza Vaccine	Self-Reported Unmet Need	Family Physician Consultation	Specialist Physician Consultation	Regular Medical Doctor
Urban - CMA^b^	0.97 (0.88, 1.06)	0.95 (0.84, 1.06)	1.11 (1.00, 1.22)	1.24 (1.15, 1.35)^d^	0.77 (0.64, 0.92)^d^
Urban - CA^e^	1.00	1.00	1.00	1.00	1.00
Rural - Strong MIZ	0.93 (0.84, 1.03)	0.85 (0.75, 0.97)^c^	0.99 (0.89, 1.11)	1.04 (0.95, 1.15)	0.96 (0.81, 1.13)
Rural - Moderate MIZ	0.93 (0.85, 1.02)	0.83 (0.75, 0.94)^d^	1.00 (0.90, 1.11)	0.91 (0.84, 1.00)	0.89 (0.76, 1.04)
Rural - Weak or No MIZ	0.89 (0.81, 0.98)^c^	0.85 (0.75, 0.96)^d^	1.01 (0.91, 1.12)	0.90 (0.82, 0.99)	0.62 (0.53, 0.74)^d^

^a ^Adjusted for chronic conditions, self-rated health status, age, marital status, educational attainment, ethnic origin, having regular medical doctor, income adequacy, having pharmaceuticals insurance, and occupation class.

^b ^CMA = Census Metropolitan Area; CA = Census Amalgamation; MIZ = Metropolitan Influence Zone

^c ^Significant at p < 0.05

^d ^Significant at p < 0.001

^e ^Reference Category

## Discussion

The results show that there is inequity in access to health care services across the rural-urban continuum, and that some of this inequity remains once other determinants are accounted for.

The finding that there is no difference in utilization of family physicians along the rural-urban continuum have been previously reported based on data from the CCHS [29], as have the findings regarding urban residents being more likely to consult a specialist physician [14,28,30,31], These findings are likely a result of supply induced demand, where the concentration of specialist physicians in urban centres and of family physicians in smaller cities brings about higher rates of specialist physician utilisation in large cities [32]. This maldistribution of physicians across the country can also explain why residents of the most urban and most rural communities were less likely to have a regular medical doctor. The lower likelihood among residents in major urban cities may also be due to the availability of drop-in health clinics meaning residents can obtain care at a time and location that is convenient rather than establishing a regular medical doctor [33].

Residents of rural communities were less likely to report having unmet health care needs. An optimistic interpretation of this finding is that rural residents are indeed having their health care needs met, however given their similar level of utilization of family physicians, lower usage of specialist physicians and poorer health status, this is unlikely. This result suggests different expectations of the health care system, leading to rural residents having a different threshold at which they report their needs being unmet. Researchers in Australia found that rural residents are more likely to postpone seeking care until economically or socially convenient which may influence their response to this question [34,35].

That a great deal of variation in access is observed between the provinces is not surprising given health care is organized and provided at the provincial level. This is particularly evident in residents of Ontario being much more likely to have received a flu shot since Ontario is the only province to have provided universal influenza vaccination coverage since the 2000-01 flu season [36,37]. The addition of place of residence to the logistic regression model causes the odds ratios for the two most rural categories to move closer to 1, leaving only the most rural category as less likely to have a flu shot.

Variation by province is also revealed in physician utilisation, with residents of Quebec and Ontario being less likely to report having consulted a family physician; residents of Quebec having a higher likelihood of consulting a specialist physician; residents of Nova Scotia have a greater likelihood of having a regular medical doctor; and residents of Quebec have a significantly reduced odds of having a regular medical doctor. Similar results have been previously reported based on data from the Canadian National Population Health Survey (1994)[31] and the CCHS (2001)[38,39].

The results from Quebec are different from the rest of the country with residents having a greater odds of seeing a specialist physician and a lower odds of seeing a family physician, they are also less likely to have a regular medical doctor. It appears as though residents of Quebec are permitted to see a specialist physician without a referral from a family physician which is different from the rest of the country where a referral is required in order to see a specialist physician. It has also been suggested that Quebecers receive more care from community health clinics and so do not report having a regular medical doctor [33].

The CCS, or municipality, of residence is attributed with a moderate level of variation in each of the physician related outcomes, most marked being having a regular medical doctor. Municipality of residence plays a role because it is at this level at which health care resources are immediately available. People in a given community share the same geographic/physical barriers to seeking care. Community level characteristics that may influence this result include physician supply, average neighbourhood income, ethnic composition, unemployment rates, income inequality, supply of community health centres, percentage of non-citizens, or levels of educational attainment [40-47]. Variation in unmet need by community may be related to resources that are available within the community. It could also be associated with expectations of the health care system which are defined in part at a community level. The addition of place of residence to the logistic regression models reduces the observed differences along the rural-urban categories and in a few cases it moves categories from showing a statistically significant difference to no differences.

Overall the small contribution from health region to each of the five outcomes is likely due, in part, to the specific survey measures being used. Although a good deal of health care services administration and planning occurs at the health region level, this is usually related to acute and long term care facilities and community care functions. In general, physician reimbursement and workforce planning occurs at the provincial level. It may also be a result of the health regions being too large and heterogeneous to reflect any place effect. A study previously done in the province of Ontario found that health regions accounted for less than 1 percent of variation in health outcomes, and that smaller jurisdictional areas reflected more of a place effect [48].

The limitations of this study are largely related to the design and conduct of the Canadian Community Health Survey (CCHS). Because the study is based on survey data there is a risk of a recall bias: respondents are asked to remember their utilization in the previous twelve months, and previous two years in the case of influenza vaccines. There are limitations related to the target sample of the survey, most notably the exclusion of a large number of Aboriginal Canadians through exclusion of Indian reserves, Crown land, and very remote areas. This may result in overestimating the level of access in rural areas.

The results of this study update the literature on access to health care services across the rural-urban continuum while controlling for other factors that are associated with access to care. The main strengths of this study are the use of data from a large nationally representative survey which allows for the identification of variation that might not otherwise be detected; the use of multilevel logistic modelling techniques to account for place of residence at a number of nested levels; and using the Statistical Area Classification to identify rural and urban status in six categories which provides greater sensitivity.

## Conclusions

This study highlights some areas where there is inequity in access and utilisation of care. The greatest degree of inequity is in utilisation of specialist physician services with those who reside in major urban centres or the province of Quebec being more likely to have consulted a specialist. The observed differences in self-reported unmet need present interesting questions about expectations. Further work needs to be done to understand rural residents expectations and their interpretation of the survey questions. Variation of effects across municipalities is an important area for further study and should include factors such as physician supply; travel distance required for health care; and socio-economic factors such as community income levels and proportion of the population that is Aboriginal.

Based on the findings of this study, researchers who are evaluating potential inequities in access, should at the very least include province or region of the country as a variable associated with access; and if systematic variation by health region or municipality is plausible, place of residence at these levels should also be accounted for.

An important message of this paper for health care policy makers is that despite universal health insurance coverage, inequities in access to care still exists between rural and urban residents. These inequities can be masked when evaluation is done at a very large scale with gross indicators of rural-urban and when the determinants of access, including place of residence are not taken into account. Understanding the relationship between rural-urban and other determinants will help policy makers to target interventions appropriately: to specific demographic, provincial, community, or rural categories.

## List of Abbreviations

CA: Census Agglomerations; CCHS: Canadian Community Health Survey; CCS: Consolidated Census Subdivisions; CHA: Canada Health Act; CI: Confidence Interval; CMA: Census Metropolitan Areas; CSD: Census Subdivisions; HBM: Health Behaviour Model; ICC: Intraclass Correlation Coefficients; MIZ: Metropolitan Influenced Zone; MOR: Median Odds Ratio; SAC: Statistical Area Classification.

## Competing interests

The authors declare that they have no competing interests.

## Authors' contributions

LS conceived of the study, participated in its design and coordination, performed the statistical analysis and drafted the manuscript. JW participated in the design of the study and helped to draft the manuscript. Both authors read and approved the final manuscript.

## Pre-publication history

The pre-publication history for this paper can be accessed here:

http://www.biomedcentral.com/1472-6963/11/20/prepub

## Supplementary Material

Additional File 1 **Appendix B - Multilevel Logistic Regression Models for All Outcomes**. This document contains the odds ratios for all of the independent variables that were controlled for in the fully adjusted multilevel logistic regression models for each of the five access measures. Click here for file
