# Supplementary material for: An evaluation of access to health care services along the rural-urban continuum in Canada
Source: BMC Health Serv Res. 2011 Jan 31;11:20. doi: 10.1186/1472-6963-11-20 (PMC3045284; doi:10.1186/1472-6963-11-20)
Supplement: Additional File 1 — Appendix B - Multilevel Logistic Regression Models for All Outcomes. This document contains the odds ratios for all of the independent variables that were controlled for in the fully adjusted multilevel logistic regression models for each of the five access measures. [file 1472-6963-11-20-S1.PDF]

Appendix: Multilevel Logistic Regression Models for All Outcomes

Odds Ratio (95% CI) Higher numbers indicate a greater odds of the outcome.

Variable	Influenza Vaccine	Self-Reported Unmet Need	Family Physician Consultation	Specialist Physician Consultation	Regular Medical Doctor
**Rural/Urban Status**					
**Statistical Area Classification** [a]					
- Urban - CMA	0.97(0.89,1.06)	0.95(0.84,1.06)	1.11(1.00,1.22)	1.24(1.15,1.35) [c]	0.77(0.64,0.92) [c]
- Urban - CA [d]	1.00	1.00	1.00	1.00	1.00
- Rural - Strong MIZ	0.93(0.84,1.03)	0.85(0.75,0.97) [b]	0.99(0.89,1.11)	1.04(0.95,1.15)	0.96(0.81,1.13)
- Rural - Moderate MIZ	0.93(0.85,1.02)	0.83(0.74,0.94) [c]	1.00(0.90,1.11)	0.91(0.84,1.00)	0.89(0.76,1.04)
- Rural - Weak or No MIZ	0.89(0.81,0.98) [b]	0.85(0.75,0.96) [c]	1.01(0.91,1.12)	0.90(0.82,0.99)	0.62(0.53,0.74) [c]
**Need**					
**Chronic Conditions**					
- None	0.72(0.69,0.75) [c]	0.74(0.70,0.79) [c]	0.53(0.51,0.56) [c]	0.62(0.59,0.65) [c]	0.52(0.49,0.55) [c]
- 1 chronic condition [d]	1.00	1.00	1.00	1.00	1.00
- 2 or more conditions	1.48(1.42,1.54) [c]	1.65(1.56,1.76) [c]	1.65(1.55,1.75) [c]	1.58(1.52,1.65) [c]	1.60(1.48,1.72) [c]
**Self-rated Health**					
- Excellent	0.86(0.83,0.90) [c]	0.45(0.42,0.48) [c]	0.66(0.63,0.69) [c]	0.64(0.62,0.67) [c]	1.03(0.97,1.08)
- Very good	0.93(0.89,0.96) [b]	0.67(0.64,0.70) [c]	0.86(0.82,0.90) [c]	0.80(0.77,0.83) [c]	1.11(1.05,1.16) [c]
- Good [d]	1.00	1.00	1.00	1.00	1.00
- Fair	1.11(1.05,1.17) [c]	1.61(1.50,1.72) [c]	1.22(1.13,1.31) [c]	1.64(1.55,1.72) [c]	1.21(1.10,1.33) [c]
- Poor	1.24(1.13,1.37) [c]	3.39(3.07,3.74) [c]	1.59(1.35,1.86) [c]	2.81(2.57,3.07) [c]	1.39(1.15,1.68) [c]

Appendix: Multilevel Logistic Regression Models for All Outcomes

Odds Ratio (95% CI) Higher numbers indicate a greater odds of the outcome.

Variable	Influenza Vaccine	Self-Reported Unmet Need	Family Physician Consultation	Specialist Physician Consultation	Regular Medical Doctor
**Predisposing Characteristics**					
**Sex**					
- Female[d]	1.00	1.00	1.00	1.00	1.00
- Male	0.93(0.90,0.96)[b]	0.72(0.69,0.76)c	0.61(0.59,0.63)c	0.64(0.62,0.66)c	0.48(0.46,0.50)c
**Age**					
- 20 to 29 years	0.34(0.32,0.36)[c]	1.14(1.06,1.23)[c]	0.47(0.44,0.50)[c]	0.61(0.58,0.65)[c]	0.19(0.17,0.20)[c]
- 30 to 39 years	0.56(0.53,0.59)[c]	1.79(1.68,1.92)[c]	0.98(0.93,1.04)	1.16(1.10,1.22)[c]	0.52(0.49,0.56)[c]
- 40 to 49 years	0.67(0.64,0.70)[c]	1.43(1.34,1.52)[c]	0.93(0.88,0.98)[b]	0.96(0.92,1.01)	0.77(0.72,0.82)[c]
- 50 to 59 years[d]	1.00	1.00	1.00	1.00	1.00
- 60 to 69 years	2.01(1.90,2.12)[c]	0.68(0.62,0.74)[c]	1.16(1.08,1.25)[c]	1.04(0.99,1.10)	1.61(1.45,1.79)[c]
- 70 to 79 years	4.14(3.86,4.44)[c]	0.52(0.47,0.58)[c]	1.34(1.22,1.47)[c]	0.91(0.85,0.97)[b]	2.23(1.94,2.56)[c]
- 80+ years	4.72(4.26,5.22)[c]	0.31(0.26,0.37)[c]	1.61(1.40,1.86)[c]	0.68(0.62,0.75)[c]	1.90(1.55,2.32)[c]
**Marital Status**					
- Married or equivalent[d]	1.00	1.00	1.00	1.00	1.00
- Single/divorced/widowed	1.02(0.99,1.06)	1.04(0.99,1.09)	0.84(0.81,0.88)[c]	0.91(0.88,0.94)[c]	0.67(0.64,0.70)[c]
**Educational Attainment**					
- < secondary graduation	1.01(0.96,1.06)	0.97(0.90,1.04)	0.97(0.92,1.03)	0.79(0.76,0.84)[c]	0.90(0.84,0.96)[c]
- Secondary graduation[d]	1.00	1.00	1.00	1.00	1.00
- Other post-secondary	0.95(0.89,1.01)	1.39(1.28,1.51)[c]	1.19(1.12,1.28)[c]	1.21(1.14,1.29)[c]	0.87(0.80,0.94)[b]
- Post-secondary graduation	1.17(1.12,1.22)[c]	1.30(1.23,1.38)[c]	1.24(1.19,1.29)[c]	1.26(1.21,1.31)[c]	0.94(0.89,0.99)[c]
**Ethnic or Cultural Origin**					
- White[d]	1.00	1.00	1.00	1.00	1.00
- Not White	1.17(1.11,1.22)[c]	0.94(0.89,1.00)	0.90(0.86,0.95)[c]	0.74(0.71,0.77)[c]	1.15(1.08,1.22)[c]

Appendix: Multilevel Logistic Regression Models for All Outcomes

Odds Ratio (95% CI) Higher numbers indicate a greater odds of the outcome.

Variable	Influenza Vaccine	Self-Reported Unmet Need	Family Physician Consultation	Specialist Physician Consultation	Regular Medical Doctor
**Enabling Resources**					
**Has a Regular Medical Doctor**					
- Yes[d]	1.00	1.00	1.00	1.00	
- No	0.50(0.48,0.53)[c]	1.66(1.57,1.76)[c]	0.23(0.22,0.24)[c]	0.66(0.63,0.69)[c]	
**Income Adequacy (2 cats)**					
- Low income	0.84(0.79,0.89)[c]	1.19(1.10,1.28)[c]	0.95(0.89,1.01)	0.92(0.87,0.97)[b]	0.68(0.63,0.73)[c]
- Middle or High income[d]	1.00	1.00	1.00	1.00	1.00
- unknown, NA, or not stated	0.99(0.94,1.04)	0.92(0.85,0.99)	1.00(0.94,1.07)	0.84(0.80,0.89)[c]	0.95(0.88,1.02)
**Has Pharmaceuticals Insurance**					
- Yes[d]	1.00	1.00	1.00	1.00	1.00
- No	0.73(0.70,0.76)[c]	1.22(1.16,1.28)[c]	0.74(0.71,0.77)[c]	0.80(0.77,0.83)[c]	0.65(0.62,0.68)[c]
**Occupation Class**					
- Bus./soc. services /hlth care [d]	1.00	1.00	1.00	1.00	1.00
- Sales and service	0.77(0.74,0.80)[c]	0.96(0.91,1.02)	0.89(0.85,0.93)[c]	0.86(0.82,0.90)[c]	1.06(1.01,1.13)
- Trades/transport/primary	0.70(0.66,0.73)[c]	0.99(0.93,1.06)	0.85(0.81,0.89)[c]	0.80(0.76,0.84)[c]	0.91(0.86,0.96)[b]
- Not Employed	0.96(0.91,1.00)	0.84(0.78,0.89)[c]	0.83(0.78,0.87)[c]	1.00(0.95,1.05)	0.95(0.89,1.02)

Appendix: Multilevel Logistic Regression Models for All Outcomes

Odds Ratio (95% CI) Higher numbers indicate a greater odds of the outcome.

Variable	Influenza Vaccine	Self-Reported Unmet Need	Family Physician Consultation	Specialist Physician Consultation	Regular Medical Doctor
**Place of Residence**					
**Province**					
- British Columbia[d]	1.00	1.00	1.00	1.00	1.00
- Alberta	0.84(0.72,0.97)[b]	0.82(0.66,1.01)	0.87(0.73,1.04)	0.86(0.74,0.99)	0.67(0.49,0.92)[c]
- Saskatchewan	0.81(0.68,0.95)[c]	0.72(0.57,0.91)[c]	0.87(0.72,1.05)	1.02(0.87,1.19)	0.73(0.54,0.99)
- Manitoba	0.59(0.49,0.71)[c]	1.02(0.80,1.30)	0.82(0.66,1.01)	0.95(0.80,1.13)	0.58(0.41,0.81)[c]
- Ontario	1.66(1.48,1.87)[c]	0.86(0.73,1.02)	0.67(0.58,0.77)[c]	1.08(0.97,1.20)	1.38(1.06,1.80)[c]
- Quebec	0.61(0.55,0.69)[c]	0.98(0.83,1.16)	0.52(0.45,0.60)[c]	1.40(1.25,1.56)[c]	0.43(0.33,0.56)[c]
- New Brunswick	0.67(0.57,0.80)[c]	0.94(0.74,1.19)	0.83(0.68,1.01)	1.17(0.99,1.37)	1.31(0.91,1.89)[c]
- Nova Scotia	1.10(0.91,1.33)	0.91(0.70,1.18)	0.89(0.71,1.12)	1.09(0.91,1.30)	2.19(1.43,3.37)[c]
- Prince Edward Island	0.74(0.55,1.01)	0.62(0.40,0.97)[b]	1.07(0.75,1.52)	1.14(0.86,1.52)	1.40(0.79,2.51)
- Newfoundland	0.48(0.39,0.59)[c]	1.02(0.78,1.33)	1.17(0.93,1.47)	1.07(0.89,1.29)	0.56(0.38,0.81)[c]
**Health Region**					
- Median Odds Ratio (ICC)[e]	1.10 (0.24%)	1.19 (0.33%)	1.13 (0.27%)	1.09 (0.16%)	1.34 (1.14%)
**Consolidated Census Subdivision**					
- Median Odds Ratio (ICC)[e]	1.32 (1.89%)	1.38 (1.16%)	1.41 (2.19%)	1.26 (1.21%)	1.88 (5.05%)

[a] CMA=Census Metropolitan Area; CA=Census Amalgamation; MIZ=Metropolitan Influence Zone

[b] Significant at $p < 0.05$

[c] Significant at $p < 0.001$

[d] Reference Category

[e] Median Odds Ratio – Interpreted as the expected increased odds if an individual moves to a different area, while all other factors remained the same. The Intraclass Correlation Coefficient (ICC) represents the proportion of the variation that is attributable to the specified
